# Dental Education as Connected with Dental Associations

**Published:** 1866-06

**Authors:** S. D. Stewart


### DENTAL EDUCATION AS CONNECTED WITH DENTAL ASSOCIATIONS.

BY DR. S. D. STEWART.

Read before the Central Ohio Dental Association.

"That which is worth doing at all is worth doing well." A hoary axiom, yet how true. How just in all its bearings and signification in relation to all the various and multiplied vocations of man. Equally just and true is it, whether applied to the ruler or the subject, the philosopher or politician, the mechanic or professional man. Everything, to give just remuneration and satisfaction to the parties concerned *should* be done well; as well as intelligence, a sense of justness, and a conscience will dictate. And in what manner and by what means can the justness and signification of the proverb be attained?

To be truly successful is to "do a thing well;" to "do a think well" is to fully understand the vocation adopted; to fully understand *any* legitimate vocation, is to be educated thoroughly in that vocation. Therefore, education is a necessary and indispensable attribute or concomitant to success in any vocation. And (Mr. President and brothers) is it not true professional success we aim at? Should it not be our goal? Is it not education, then, we so much need? Or are there members of our fraternity who have reached the climax of learning, and are perfect? The man who cannot always learn, although he be considered one of the "leading lights" of his profession, needs our sympathy. He will not long be a "leading light," if he does not keep pace with the advancing knowledge and improvements of his profession. Is it not education, *continued* education we so much need? Dental education? In what way can we more profitably and pleasantly pass a few hours or days; in what way can we most cultivate that feeling of friendship and friendly emulation, stimulants to progress and advancement? In what way can we be made to be co-workers together for the advancement of the profession we represent, and be made to feel that we have a common interest in its advancement, and upward growth, than by meeting together and discussing in a friendly and courteous manner the various topics that pertain to the elevation of the profession and our own interests personally? I say in what way can it be better accomplished and more easily attained than by encouragement and free support to the associations forming, as well as those which are formed? We meet as an association for what? To accomplish in part at each session that for which the association was formed. It will be purely beneficial in its results to each member individually and to the members collectively if sustained and encouraged in the right spirit. It will do away with cold hands and hearts, frowning faces and the passing by of each other without the proper salutation; and instead thereof we will have the warm friendly grasp and welcome; the smiling face and brotherly salutation when we meet.

An association of a true character *does not* encourage a feeling of enmity and hate, and uncharitable rivalry, the rightful subjects of ignorance; but on the contrary we meet as brothers having a common interest at stake; entertain a more charitable feeling, and emulate each other in the spirit we should. The principle object or duty of an association then is to EDUCATE, to INSTRUCT. Then may we not encourage and freely support Dental Associations? In so doing we encourage the most important element of success. For without education what would we be as a profession? What would we be as a nation? Education stimulates to higher and nobler acts, and purer motives; it elevates the savage to a state of civilization; the empiric to that of a true professional man.

Dental education will elevate the profession to that of its kindred professions. With it, it will soar in its true sphere; without it, it will as a natural consequence of darkness, relaps, or stand in that state in which it should, that of an ignoble profession.

We do not want to be cobling or tinkering, as it were, at our profession, but we want to make rapid strides, and speedily become a recognized and a true profession. We weary of being goaded on, we have a dislike to be stirred up so often upon one thing, and yet at the same time that one thing may be the "*one thing needful*" the only weak point of our great enemy—ignorance—and through whose lines, by a stragetic movement we may break and come out victorious, and the title "D.D.S." (apparently) mean more and shine brighter than ever before. And yet we hate to be stirred up and will *not* give the support we should to the *only things* that will strengthen and give us the victory. Truly, truly, we are standing in our own light, we grasp at the shadow not the substance. If we do not follow the bright beacons far ahead the result will be direful. If we do not add strength to the links of the great chain encircling and supporting our profession it will become weak, and like a rope of sand, fall of its own weight. Let strength be added to each link that it may become as a chain of steel—inseperable. Let each link added to the still increasing chain be purified and a brighter one; let it be like unto a chain of gold, "*purified in a furnace of fire seven times.*"

In view of these facts, then, are we not *all* ready to admit that Dental Associations *educate* and act as fulcrums to that powerful lever which alone can raise us to that high standard upon which all true workers will rejoice to see us placed?

Dental Associations may be the *alpha* of our education, but it has no *omega*. When the title of "Doctor-of-Dental Surgery" has reached its highest signification in a practical as well as literal sense, may the memory of those who have been so instrumental in their labors to attain that end, in the language of the Poet:—

> Flourish in immortal youth,
> Unhurt amid the war of elements,
> The wreck of matter, and the crash of worlds.

